# Out of the Cold: Does the Immunoproteasome Play a Role in Kidney Graft Function after Cold Storage?

**DOI:** 10.34067/KID.0000000000000436

**Published:** 2024-05-30

**Authors:** Paul M. O'Connor

**Affiliations:** Department of Physiology, Augusta University, Augusta, Georgia

**Keywords:** immunology, kidney transplantation, renal ischemia, renal transplantation

In 1954, the kidney was the first solid organ to be transplanted successfully between humans in genetically identical twins.^1^ By the 1960s, infusion of cold solution into the renal artery of the harvested kidney was established.^1^ This greatly increased the time in which kidneys could be stored and successfully transplanted. Along with advances in tissue matching and immunosuppression, cold preservation has enabled the storage and transport of donor kidneys that underlies our current kidney transplant network, enabling tens of thousands of life-saving kidney transplants annually in the United States alone.

Kidney ischemia is an unavoidable consequence of the transplant process. Ischemic injury may occur before harvesting the kidneys from cadaveric donors, in which the circulation has stopped, or during organ harvest itself. Despite preservation of the harvested kidneys using cold storage, ischemic injury continues to occur, albeit at a slower rate, while the organ is being stored or transported. Finally, surgical implantation and reperfusion of the donor kidney in the recipient may lead to further ischemic injury.^2^ This injury is thought to be associated with reperfusion of the ischemic donor kidney with oxygenated blood, which results in the excess production of reactive oxygen species and release of inflammatory mediators. Critically, ischemic injury to the kidney during the transplant process is known to significantly worsen clinical outcomes, with cold ischemic time being an independent risk factor of both graft failure and delayed graft function.^2,3^ As such, prolonged cold storage of kidneys often results in donor kidneys being discarded.

The concept behind the beneficial effects of cold storage is that lowering the temperature of the donor kidney slows its metabolism, protecting the organ from the deleterious effects of energy depletion. While cellular energy depletion undoubtably disrupts many cellular functions, the molecular mechanisms mediating kidney injury from cold storage are not entirely understood. Identification of these mechanisms has the potential to lead to new therapies to further improve kidney preservation and outcomes for patients in need of a kidney transplant.

In this issue of *Kidney360*, Bhattarai *et al.*^4^ investigate the role of the immunoproteasome in mediating the deleterious effects of cold storage on kidney allograft function. The proteasome is a barrel-shaped protein complex that is involved in the degradation of ubiquitinated intracellular proteins into small peptide fragments. Most cells express what is called the constitutive proteasome. Bhattarai *et al.* had previously demonstrated that the peptidase activity of the constitutive proteasome was decreased in kidney cells after cold storage and that this led to reduced graft function, potentially by impairing mitochondrial function.^5–7^ The immunoproteasome differs from the constitutive proteasome in that several of the constitutively expressed catalytic *β* subunits of the standard proteasome are replaced by immunoproteasome-specific inducible counterparts.^8^ These include the immunoproteasome-specific subunits LMP2 for constitutive *β*1, LMP10 for *β*2, and LMP7 for *β*5.^8^ While the immunoproteasome is typically active in immune cells, after injury or inflammation, it can also be activated in nonimmune cells by transcription of these immunoproteasome-specific subunits.^8^ In damaged tissue, this occurs in response to reactive oxygen species and inflammatory cytokines, two factors known to be critical in cold storage/transplant injury.^6^ The immunoproteasome differs functionally from the constitutive proteasome in that it produces peptides with a hydrophobic C terminus that can be presented by MHC class I molecules on the cell surface. The display of peptide fragments by MHC-1 is then surveilled by CD8^+^ T cells and plays an important role in activation of the adaptive immune system. While inhibition of the immunoproteasome has been reported to be protective against ischemia-reperfusion injury in the heart^9^ and brain^10^, the role of the immunoproteasome in cold storage–induced kidney injury remains largely unknown.

In their *Kidney360* article,^4^ Bhattarai *et al.* investigate the role of the immunoproteasome in cold storage/transplant-induced graft injury using two rat kidney transplant models. One was a syngenetic transplant model in which Lewis rats were used as both the donor and recipient strains. The other was an allogenic model in which Fischer rats were used as donors while Lewis rats served as the recipient strain. In both cases, donor rat kidneys were flushed with Wisconsin solution and stored at 4°C (cold storage) for either 4 or 18 hours before surgical attachment of the donor kidney. To assess the role of the immunoproteasome in graft function, ONX0914, a specific and reversible inhibitor of the LMP7 or *β*5_i_ catalytic subunit of the immunoproteasome, was added to the cold storage solution. After cold storage, donor kidneys were transplanted into recipient rats by end-to-end anastomosis of the recipient and donor renal artery, vein, and ureter. Auto transplant without cold storage was used to control the effects of the transplant surgery alone while sham rats that did not undergo the transplant surgery were also used for comparison. All recipient rats underwent a uninephrectomy at the time of transplant so that renal function of the donor kidney was reflected in systemic measurements of kidney function.

The authors first major finding was that cold storage-induced expression of the LMP7 or *β*5_i_ subunit of the immunoproteasome and that subunits catalytic activity in renal tissue homogenates was also increased.^4^ Importantly, the authors found that *β*5_i_ function was persistently increased to at least 9 days post-transplant, in both syngeneic and allogenic models after both 4 and 18 hours of cold storage.^4^ The expression of the immunoproteasome multimeric enzyme complex (i26S/30S) was also increased in the kidney 9 days after transplant after both 4 and 18 hours of cold storage.^4^ Interestingly, the immunoproteasome complex was decreased after cold storage alone, absent transplantation of the kidney. This suggests that the upregulation of the immunoproteasome complex in the donor kidney occurs in response to transplantation and reperfusion of the donor kidney after cold storage rather than cold storage itself.

Next, the authors asked the question, “Does inhibiting the immunoproteasome during cold storage improve kidney function post-transplant?” To do this, ONX0914 was added to the cold storage solution. Disappointingly, inhibition of the *β*5_i_ subunit of the immunoproteasome during cold storage did not significantly improve tubular injury score, plasma creatine levels, or BUN levels in rats 9 days after syngenetic transplant donor kidneys cold-stored for 4 hours.^4^

While inhibiting the immunoproteasome during cold storage would have been the most practical approach, its failure to improve kidney outcomes cannot exclude a role of the immunoproteasome in mediating kidney cold storage injury after transplant. In fact, the author's own data suggest that upregulation of the immunoproteasome does not occur until after surgical implantation of the donor kidney.^4^ Given the persistent upregulation of the immunoproteasome post-transplant,^4^ could inhibition of the immunoproteasome during this time improve kidney function? Certainly, the upregulated immunoproteasome seems a tantalizing therapeutic target which could link cold storage–induced tissue reactive oxygen species production with the post-transplant immune response. This prospect is made even more tantalizing by evidence that systemic addition of ONX0914 post-transplant ameliorates chronic antibody-mediated rejection.^11^ As Bhattarai *et al.* point out,^4^ further studies are needed to address this critical question. Other questions also remain. Where in the kidney is the immunoproteasome overexpressed? Does upregulation of the immunoproteasome simply reflect the infiltration of immune cells into the cold storage–damaged kidney? Is the immunoproteasome upregulated in the kidney parenchyma itself, perhaps due to increased reactive oxygen species production or inflammatory cytokine signaling from damaged tubular cells? Clearly more studies are needed. By identifying upregulation of the immunoproteasome in the donor kidney post–cold storage transplant, Bhattarai *et al.* have identified a potentially powerful therapeutic target to improve kidney outcomes post-transplant.

## References

[1] Barker CF Markmann JF. Historical overview of transplantation. Cold Spring Harb Perspect Med. 2013;3(4):a014977. doi:10.1101/cshperspect.a014977 23545575 PMC3684003

[2] Ponticelli C Reggiani F Moroni G. Delayed graft function in kidney transplant: risk factors, consequences and prevention strategies. J Pers Med. 2022;12(10):1557. doi:10.3390/jpm12101557 36294695 PMC9605016

[3] Bronzatto EJ da Silva Quadros KR Santos RL Alves-Filho G Mazzali M. Delayed graft function in renal transplant recipients: risk factors and impact on 1-year graft function: a single center analysis. Transplant Proc. 2009;41(3):849–851. doi:10.1016/j.transproceed.2009.02.004 19376369

[4] Bhattarai D Lee SO Joshi N, . Cold storage followed by transplantation induces immunoproteasome in rat kidney allografts: inhibition of immunoproteasome does not improve function. Kidney360. 2024;5(5):743–752. doi:10.34067/KID.0000000000000368 38303110 PMC11146655

[5] Bhattarai D Lee SO MacMillan-Crow LA Parajuli N. Normal proteasome function is needed to prevent kidney graft injury during cold storage followed by transplantation. Int J Mol Sci. 2024;25(4):2147. doi:10.3390/ijms25042147 38396827 PMC10888692

[6] Lo S MacMillan-Crow LA Parajuli N. Renal cold storage followed by transplantation impairs proteasome function and mitochondrial protein homeostasis. Am J Physiol Renal Physiol. 2019;316(1):F42–F53. doi:10.1152/ajprenal.00316.2018 30303714 PMC6383197

[7] Lo SB Blaszak RT Parajuli N. Targeting mitochondria during cold storage to maintain proteasome function and improve renal outcome after transplantation. Int J Mol Sci. 2020;21(10):3506. doi:10.3390/ijms21103506 32429129 PMC7279041

[8] Kniepert A Groettrup M. The unique functions of tissue-specific proteasomes. Trends Biochem Sci. 2014;39(1):17–24. doi:10.1016/j.tibs.2013.10.004 24286712

[9] Sula Karreci E Fan H Uehara M, . Brief treatment with a highly selective immunoproteasome inhibitor promotes long-term cardiac allograft acceptance in mice. Proc Natl Acad Sci U S A. 2016;113(52):E8425–E32. doi:10.1073/pnas.1618548114 27956634 PMC5206568

[10] Chen X Zhang X Wang Y, . Inhibition of immunoproteasome reduces infarction volume and attenuates inflammatory reaction in a rat model of ischemic stroke. Cell Death Dis. 2015;6(1):e1626. doi:10.1038/cddis.2014.586 25633295 PMC4669779

[11] Li J Basler M Alvarez G Brunner T Kirk CJ Groettrup M. Immunoproteasome inhibition prevents chronic antibody-mediated allograft rejection in renal transplantation. Kidney Int. 2018;93(3):670–680. doi:10.1016/j.kint.2017.09.023 29229189

